# Effects of Betaine-Biotin-Chromium Supplementation and Concentrate to Roughage Ratio on Nutrient Utilization Efficiency in Thai Native Cattle

**DOI:** 10.3390/ani11092747

**Published:** 2021-09-20

**Authors:** Sukanya Poolthajit, Wuttikorn Srakaew, Theerachai Haitook, Chalong Wachirapakorn

**Affiliations:** 1 Department of Animal Science, Faculty of Agriculture, Khon Kaen University, Khon Kaen 40002, Thailand; Spx_nu177@hotmail.com (S.P.); theerachai.anisci@gmail.com (T.H.); 2 Department of Animal Science and Fisheries, Faculty of Agricultural Sciences and Technology, Rajamangala University of Technology Lanna, Nan 55000, Thailand; esso.wutt@gmail.com

**Keywords:** betaine, biotin, chromium, nutrient utilization efficiency, Thai native cattle

## Abstract

**Simple Summary:**

Feed additives are attracting considerable interest due to the need to effectively improve the level of production, efficiency, and health of animals. A combination of betaine, biotin, and chromium has been developed to enhance nutrient utilization efficiency. Ideally, the addition of a combination of supplements to livestock feed could improve ruminal fermentation, digestibility and nutrient utilization, nitrogen and energy balance, and metabolism.

**Abstract:**

The objective of this study was to evaluate the effects of supplementation with a feed additive containing a combination of betaine, biotin, and chromium (BBC) and concentrate-to-roughage ratio (C:R ratio) on nutrient digestibility, blood metabolites, ruminal fermentation, nitrogen balance, and energy utilization in Thai native beef cattle. Five Thai feedlot native beef bulls at one year old of age and 87.6 ± 15.7 kg of body weight were assigned in a (2 × 2) + 1 augmented factorial experiment according to a 5 × 5 Latin square design with five periods of 21 days. The two levels of BBC were 3 g/kg DM and 6 g/kg DM, and the two ratios of C:R were 60C:40R and 70C:30R. A C:R ratio of 70C:30R without BBC supplementation was used as a negative control. The cattle were offered feed at 3% BW, which provides sufficient energy and protein to support a weight gain of 500 g/day. The results showed that dry matter intake (DMI) and organic matter intake (OMI) were not different (*p* > 0.05), but the intake of crude protein (CP) and ether extract (EE) was higher (*p* < 0.05), whereas neutral detergent fiber (NDF) and acid detergent fiber (ADF) were lower (*p* < 0.05) in the 70C:30R ratio diets compared to the 60:40R ratio diets. Energy balance and nitrogen balance were not influenced (*p* > 0.05) by the C:R ratio or the addition of BBC. Rumen pH and ammonia-nitrogen (NH_3_-N) did not differ (*p* > 0.05) among treatments groups. Total volatile fatty acid (VFA) production was higher (*p* < 0.05) in bulls fed with the 70C:30R ratio diet. The addition of BBC resulted in a lower (*p* < 0.01) glucose-to-insulin ratio compared to cattle fed with the control diet. Energy and protein utilization efficiency did not differ (*p* > 0.05) among the experimental treatment groups, which were higher (*p* > 0.05) than the control group. In conclusion, BBC supplementation showed greater growth performance of cattle compared to the control. BBC supplementation appeared to improve glucose uptake and insulin sensitivity and energy and protein utilization efficiency. Whether BBC supplementation increases glucose production in the liver remains to be determined in future studies.

## 1. Introduction

Modern beef cattle feeding requires the optimal concentrate-to-roughage ratio (C:R ratio), which affects weight gain and the efficiency of weight gain. Ideally, a proper C:R ratio can maximize weight gain and the fattening rate with low digestive disorders [1]. Though it can range from 50:50 to 90:10, a C:R ratio of 75:25 offers reasonable weight gain with minimal risk in most feedlots. Wanapat et al. [2] demonstrated that a C:R ratio of 60:40 improved energy intake, rumen fermentation, and microbial protein production in crossbred Holstein Friesian dairy steers given urea-treated rice straw as a roughage source. To date, many studies have been published that describe novel feeding methods for beef cattle farmers, incorporating various vitamins and minerals aimed towards improving efficiency in beef cattle farming. Feed additives are widely used and can effectively improve the level of production, efficiency, and health of the animals. Feed additives are appropriate in cattle finishing operations and are more often used in farms. However, the main effect of feed additives is increasing feed efficiency and/or the average daily weight gain.

Biotin is a cofactor of propionyl CoA carboxylase (PC) and pyruvate carboxylase [3] and seems to support gluconeogenesis at the intermediate metabolism level [4]. Ferreira and Weiss [5] found that biotin supplementation affected PC activity in the liver of lactating dairy cows. The supply of glucose to the mammary gland is a major determinant of milk yield in lactating dairy cows; therefore, the increase in milk production observed in lactating dairy cows the diets of which were supplemented with biotin might be related to an increase in gluconeogenesis, presumably in the liver. Research in dairy cows has shown a consistent response to biotin supplementation (20 mg/day), including improved digestibility and a higher milk yield, which is likely associated with the tendency of high grain-based rations to compromise ruminal biotin synthesis [6]. If glucose synthesis is high, it is necessary to boost glucose uptake into the cells. Chromium is a primary determinant of glucose tolerance factor (GTF) and the maintenance of glucose homeostasis [7], which improves insulin binding and increases glucose clearance [8]. Heifers fed with a chromium-supplemented diet produce less insulin and clear glucose at a faster rate than those not supplemented with chromium [8,9]. Kegley et al. [10] and Bernhard et al. [11] reported that steer feeders fed with a chromium-supplemented diet during the exposure period had higher insulin levels and higher insulin-to-glucose ratios compared with those without additional chromium. In non-ruminants, chromium supplementation differentially impacts certain blood metabolites, including glucose [12]. In contrast, Kneeskern et al. [13] found that the addition of chromium (3 mg/day) did not improve measures of insulin sensitivity over time and had no effects on growth performance. Another common feed additive is betaine, which improves protein utilization, likely by providing the methyl groups required for the formation of 5-methyltetrahydrofolate, as well as for the regeneration of methionine in the homocysteine cycle. Supplementation with betaine in dairy cows seems to improve the milk yield [14] and the performance of dairy cows under heat stress [15]. Zhang et al. [16] found that betaine supplementation at 15 g/day increases the feed intake, milk yield, and levels of lactose and protein in the milk of dairy cows. Furthermore, Wang et al. [17] found that adding betaine at 0.6 g/kg DM to feed stimulates growth and nutrient digestion in bulls. Based on the aforementioned research, the combinatorial use of biotin, betaine, and chromium (BBC) seems to be a promising approach towards improving feed utilization and nutrient metabolism, resulting in enhanced growth performance.

Based on the above literature, BBC supplementation in ruminant diets may enhance feed utilization efficiency and production efficiency. In our previous study [18], BBC supplementation up to 6 g/kg DM quadratically increased gas accumulation, in vitro DM degradability, the efficacy of microbial biomass production, ammonia nitrogen, total volatile fatty acid, and propionate concentration. However, the effect of BBC supplementation in Thai feedlot native beef cattle fed with various C:R ratios in their diet has not been studied yet. The hypothesis could be made that BBC could enhance ruminal fermentation, nutrient metabolism, and the growth performance of Thai feedlot native beef cattle. Therefore, the aim of the present study was to evaluate the effects of BBC supplementation on nutrient digestibility, blood metabolites, ruminal fermentation, nitrogen balance, energy utilization, and weight gain in Thai feedlot native beef cattle fed with various C:R ratios in their diet.

## 2. Materials and Methods

### 2.1. Ethics and Animal Care

The experiment procedures were approved by the Animal Ethics Committee of Khon Kaen University (Record No. AEKKU23/2562), based on the Guidelines of the Ethics of Animal Experimentation of the National Research Council of Thailand.

### 2.2. Animals, Experimental Design, and Dietary Treatments

The five animals used in this study were one-year-old, Thai native bulls with an average body weight of 87.66 ± 15.70 kg (mean ± SD). The analysis involved a (2 × 2) + 1 augmented factorial experiment with a 5 × 5 Latin square design that included five dietary treatments over five 21-day periods. The two levels of BBC were 3 g/kg DM and 6 g/kg DM, and the two ratios of C:R were 60C:40R and 70C:30R. The C:R ratio of 70C:30R without BBC supplementation was used as a negative control. All of the bulls were offered diets at 3% BW with sufficient energy and protein content to support a weight gain of 500 g/d [19]. The animals were housed individually in stalls (2.5 × 4.5 m^2^), fed at 09:00 and 17:00 each day for 14 days, and then transferred to a metabolic crate to collect feed, feces, and urine samples. Clean drinking water was provided at all times.

A total collection method was employed to collect feces and urine for five consecutive days. A total of 500 g feces from the total feces was taken daily, pooled by animals and periods, and stored at −18 °C to analyze it for its chemical and energy content. Urine was collected in 1.5-L bottles containing 6 N hydrochloric acid to prevent nitrogen loss, and 500 mL urine was taken daily, pooled by animals and periods, and stored at −18 °C for the analysis of nitrogen and energy content.

The animals were offered concentrate and roughage separately, adjusted daily to obtain a dry matter intake of 3% BW. The concentrate was formulated using the ingredients shown in Table 1. Rice straw was used as the roughage source. The amount of BBC was calculated and fed on top of the concentrate during morning feeding to achieve 3 g/kg DM and 6 g/kg DM. BBC given at 3 g/kg DM and 6 g/kg DM consisted of biotin at 0.13 and 0.25 mg/kg DM, Cr at 0.13 and 0.25 mg/kg DM, and betaine at 0.32 and 0.64 mg/kg DM, respectively. Cattle were weighed on the first and last day of each experimental period in the morning (07:30) to determine the body weight and metabolic body weight for each feeding level.

On the last day of each period, rumen fluid samples (approximately 200 mL) were taken via a stomach tube at 0, 2, and 4 h after feeding. Ruminal pH was immediately determined using a portable pH and temperature meter (HANNA Instruments HI 8424 microcomputer, Singapore). The rumen fluid was then strained through four layers of cloth to remove dust, after which 10 mL of 1 M H_2_SO_4_ was added to 90 mL of rumen fluid. The mixture was centrifuged at 16,000× *g* for 15 min, and the supernatant was removed and stored at −20 °C prior to conducting an ammonia-nitrogen (NH_3_-N) analysis using the Kjeldahl method [20]. The volatile fatty acid (VFA) concentration was determined using a gas chromatograph (GC 8890; Agilent technologies Ltd., Santa Clara County, CA, USA), equipped with a capillary column (molecular sieve 13×, 30/60 mesh, Alltech Associates Inc., Deerfield, IL, USA) as described by Cai [21].

Blood samples (about 10 mL) were drawn from the jugular vein at the same time as rumen fluid sampling. The samples were centrifuged at 500× *g* for 10 min and stored at −20 °C. Blood urea nitrogen (BUN) and blood glucose were determined using a diagnostic kit (Cobas Integra 400 Plus Analyzer, Roche Diagnostics Co., Mannheim, Germany). The insulin concentration was assessed using an insulin radioimmunoassay kit (Coat-A-Count Insulin^®^ DPC^®^, CA, USA).

Samples of the offered feed, refused feed, and feces were oven-dried at 65 °C for 72 h and then ground through a 1 mm screen. AOAC procedures [19] were used to analyze the dry feed and feces samples for dry matter (DM; methods 967.03), ash (method 942.05), ether extracts (method 920.39), and crude protein (CP; method 984.13). Neutral detergent fiber (NDF) and acid detergent fiber (ADF) contents were determined using ANKOM Daisy Incubators (ANKOM Technology, Macedon, NY, USA). The buffer was prepared according to the ANKOM Technology protocol. Urine compounds were sampled to determine nitrogen content using the Kjeldahl procedure [19]. The gross energy (GE) of the feed, feces, and urine was determined using a bomb calorimeter (IKA Calorimeter System, C 2000 basic, IKA-Werke, Staufen, Germany). Digestible energy (DE) and metabolizable energy (ME) were estimated based on energy intake and energy loss through feces, urine, and methane. Methane energy was calculated according to IPCC [22].

### 2.3. Statistical Analysis

These data were subjected to a (2 × 2) + 1 augmented factorial experiment with a 5 × 5 Latin square design and then analyzed using a general linear model (GLM) procedure [23]. The model is as follows:

Yijk = µ + αi + τj + βk + Ɛijk,

where Yijk is the observation value for cattle j receiving diet i in period of k; µ is the overall mean; αi is the animal effect (i = 1, 2, 3, 4, 5); τj is the treatment effect (j = 1, 2, 3, 4, 5); βk is the time period effect (k = 1, 2, 3, 4, 5) and Ɛijk is the residual effect. The results are presented as least square means with their associated standard errors of the mean. Differences between treatment means were tested for statistical significance using Duncan’s new multiple range test, and differences amongst means were considered statistically significant at *p* ≤ 0.05. In addition, the contrasts between the control and experimental diets and within experimental diets were assessed. Statistical significance was declared at *p* ≤ 0.05 and a trend was considered when 0.05 < *p* ≤ 0.10. 

## 3. Results

No interaction effects between C:R ratio and BBC inclusion were found (*p* > 0.05) in all of the parameters tested (data not shown).

### 3.1. Nutrient Intake and Apparent Digestibility

Table 2 illustrates feed intake and apparent digestibility in Thai feedlot native beef cattle. Total DM intake (kg/d) did not differ (*p* > 0.05) among treatments. However, DM intake, expressed as %BW and g/kg W^0.75^, was significantly different among treatments; the control treatment showed a significantly higher (*p* < 0.05) DM intake than other treatments. The concentrate intake was lower (*p* < 0.05), whereas rice straw intake was higher (*p* < 0.05) in the cattle fed with the 60C:40R ratio diet than the 70C:30R ratio diet. Likewise, the organic matter (OM) intake was not significantly different (*p* > 0.05) among the treatment groups. The intake of CP and EE were lower (*p <* 0.05), whereas the intake of NDF and ADF were higher (*p <* 0.05) in beef cattle given the 60C:40R ratio diet compared with those given the 70C:30R ratio diet. The apparent digestibility of DM, OM, EE, and NDF were not different (*p* > 0.05) between the treatment groups. However, the digestibility of CP in beef cattle fed with the 60C:40R ratio diet with BBC inclusion at 3 g/kg DM was the lowest (*p <* 0.05) among all of the treatments. In addition, the digestibility of ADF in beef cattle fed with the 60C:40R ratio diet with BBC inclusion at 6 g/kg DM was significantly higher (*p <* 0.05) than in beef cattle fed with the control diet. The difference between the control diet and the treatment diets was influenced more by the C:R ratio than BBC supplementation, which did not appear to affect intake and digestibility.

### 3.2. Ruminal Fermentation and Blood Metabolites

Neither the C:R ratio nor BBC supplementation altered (*p >* 0.05) the ruminal pH, with all values from the treatment groups (6.68–6.78) being similar to the control group (Table 3). The ruminal NH_3_-N concentration did not vary (*p >* 0.05) between the dietary treatments either. However, the total VFA in beef cattle fed with the 70C:30R ratio diet was significantly higher (*p* < 0.05) than in cattle fed with the 60C:40R ratio diet. The molar percentages of acetic acid (A), propionic acid (P), butyric acid, and the A:P ratio were not influenced (*p >* 0.05) by the C:R ratio or BBC supplementation.

**Figure 1:**
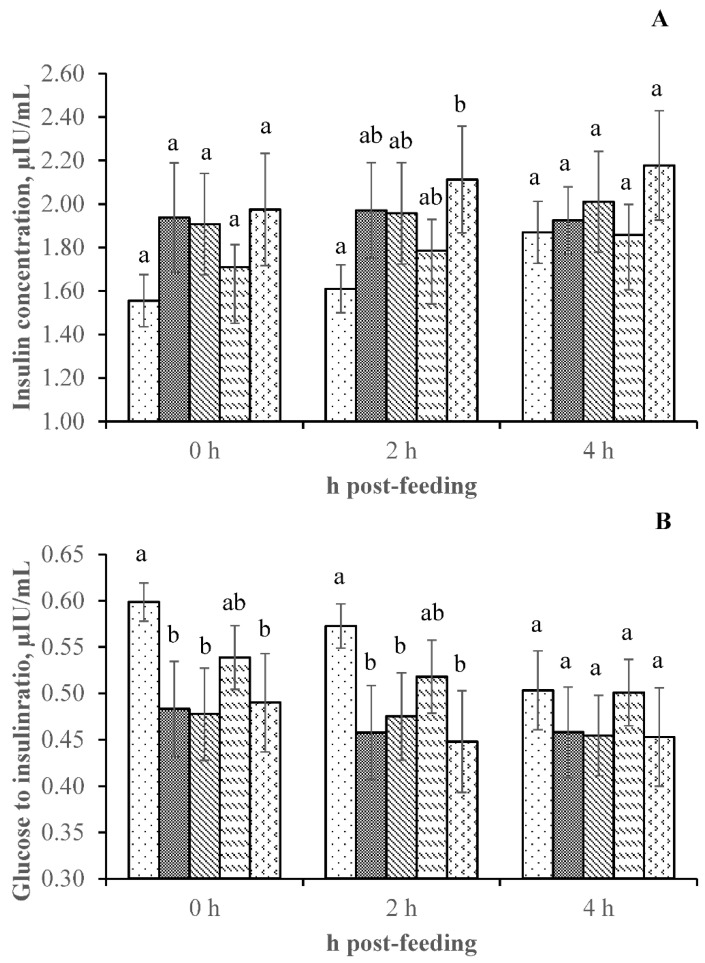
(**A**) Insulin concentration and (**B**) glucose-to-insulin ratio at 0, 2, and 4 h post-feeding in beef cattle offered dietary treatments. 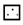 = control (70C:30R without BBC), 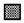 = 60C:40R with BBC at 3 g/kg, 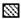 = 60C:40R with BBC at 6 g/kg, 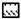 = 70C:30R with BBC at 3 g/kg, 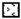 = 70C:30R with BBC at 6 g/kg. ^a,b^ Means with different superscripts were significantly different (*p* < 0.05).

The glucose-to-insulin ratio was significantly (*p* < 0.05) different between treatments, with the control treatment being significantly higher than the 60C:40R diet with 3 g/kg BBC and 6 g/kg BBC, and the 70C:30R diet with 6 g/kg BBC (Table 3 and Figure 1). Blood urea nitrogen, glucose, and insulin concentrations were not different (*p* > 0.05) between the treatment groups. Increasing C:R ratios significantly increased (*p* < 0.05) the BUN and glucose concentrations (Table 3). BBC supplementation did not affect BUN, glucose, insulin, or the glucose-to-insulin ratio. The insulin concentration in beef cattle fed with the treatment diets tended to be higher (*p* = 0.08) than in those fed with the control diet.

### 3.3. Nitrogen Balance

The nitrogen balances of the cattle fed with different C:R ratio diets and different levels of BBC are presented in Table 4. No interaction effect was found between C:R ratio and BBC inclusion in terms of nitrogen balance (*p* > 0.05). Nitrogen intake and absorption were significantly lower (*p* < 0.05) in 60C:40R at both levels of BBC supplementation compared with those of the control diet group. Fecal nitrogen was significantly (*p* < 0.05) observed between control and 60C:40R with 6 g/kg BBC supplementation. Urinary nitrogen excretion did not differ (*p* > 0.05) according to the treatment groups. Total nitrogen excretion was significantly (*p* < 0.05) lower in 60C:40R and 70C:30R diets with 6 g/kg BBC supplementation compared with the control. Increasing the C:R ratio significantly (*p* < 0.05) increased nitrogen intake, nitrogen absorption, nitrogen retention, and the efficiency of nitrogen absorption. BBC supplementation at 6 g/kg significantly (*p* < 0.05) decreased total nitrogen excretion compared with 3 g/kg BBC supplementation.

### 3.4. Energy Partitioning and Energy Efficiency

No interaction effect between C:R ratio and BBC addition was found (*p* > 0.05). Gross energy intake, fecal energy excretion, urine energy excretion, methane energy, digestible energy, and metabolizable energy were not different (*p >* 0.05) among treatments (Table 5). The ratio of DE/GE, ME/GE, and ME/DET were not different among the treatments. The C:R ratio and BBC supplementation did not appear to have any effect on energy partitioning and efficiency.

### 3.5. Weight Gain, Feed Conversion Ratio, Feed Efficiency, and Nutrient Utilization Efficiency

Weight change and gains were significantly (*p* < 0.05) higher in both C:R ratios with 6 g/kg BBC supplementation compared to the control (Table 6). The feed conversion ratio (FCR) in the control treatment was significantly higher (*p* < 0.05) than in the other treatments. The efficiency of energy and protein utilization was significantly (*p* < 0.01) lower in the control than in the other treatments. Protein intake was significantly (*p* < 0.01) lower in the 60C:40R group with BBC supplementation compared to the control. Increasing the C:R ratio had no effect on growth performance or energy utilization, with the exception of protein intake. Protein intake was significantly (*p* < 0.01) higher in 70C:30R than 60C:40R. BBC supplementation did not affect growth performance, energy utilization, or protein utilization.

## 4. Discussion

### 4.1. Nutrient Intake and Apparent Digestibility

In this experiment, C:R ratios did not influence the nutrient intake of DM and OM but did affect CP, EE, NDF, and ADF intake because there is less CP and EE and more NDF and ADF contained in diets with different C:R ratios. Furthermore, the digestibility of DM, OM, EE, and NDF was not significantly affected, whereas the digestibility of CP and ADF was affected by the different C:R diets. Nevertheless, supplementation with BBC did not affect intake and digestibility. Wanapat et al. [2] reported that increasing the proportion of concentrate from 20% to 80% in daily steers did not influence total DMI; however, an increase in concentrate has been shown to linearly increase with the digestibility of DM, OM, and CP, whereas it decreases with the digestibility of NDF and ADF. In the current study, cattle fed with the 70C:30R ratio diet showed lower (*p* = 0.06) ADF digestibility than did cattle fed with the 60C:40R ratio diet. This finding could be explained by the fact that the number of cellulolytic bacteria decreased as the proportion of concentrate in the diet increased [2,24]. Majee et al. [25] reported that biotin supplementation (20 mg/d) had no effect on the total tract digestibility of DM, OM, or NDF. Moreover, Shah et al. [26] found that supplementation with betaine (15 g/day) increased the apparent digestibility of DM, OM, CP, NDF, and ADF in dairy cows. Similarly, Deka et al. [27] reported that the apparent digestibility of DM, OM, CP, and ADF increased after supplementation with 1.5 mg Cr/kg DM in buffaloes. Productivity improvements associated with chromium supplementation have generally been observed during stress [28]. However, Kumar et al. [29] concluded that inorganic chromium supplementation in buffalo Murrah calves did not affect DM intake. Independent reports also support that dietary supplementation with chromium dose not impact the digestibility of DM, OM, and CP [30].

### 4.2. Ruminal Fermentation and Blood Metabolites

Ruminal pH was not affected by the C:R ratio or BBC inclusion, ranging between 6.68–6.78. This result is in line with that of Suksathit et al. [31], who found a range of 6.88–7.02 in Thai feedlot native cattle fed diets containing a 65% proportion of concentrate. In addition, Kongphaitee et al. [32] revealed that Thai native cattle fed with TMR containing 50% rice straw have a high ruminal pH (pH 7.0). High ruminal pH was found in diets with a high proportion of roughage [33]. Wanapat et al. [2] suggest that a roughage-to-concentrate ratio of 40:60 has positive effects on the creation of healthy rumen (ruminal pH and ecology) in dairy steers fed with urea-treated rice straw as their source of roughage. Likewise, some researchers have reported that feeding of a diet high in concentrate (67% or 60%) has no effect on mean ruminal pH or NH_3_-N concentrations [34]. Ruminal NH_3_-N (11.29–13.17 mg/dL) in the current study was in the normal range for beef cattle fed diets containing 11.15%–12.39%CP. The ruminal NH_3_-N concentration should range from 10 to 25 mg/dL, which is suitable for rumen fermentation and enhanced microbial yield in cattle [35].

In the present study, total VFA production was higher in cattle fed with the 70C:30R ratio diet than in cattle fed with the 60C:40R ratio diet but did not differ from those fed with the control diet. However, the proportion of acetic acid, propionic acid, butyric acid, and the A:P ratio did not differ. The proportion of individual VFA obtained was within the normal range when more concentrate was fed to these ruminants [24]. Furthermore, as the roughage-to-concentrate ratio decreased, the A:P ratio also decreased [34]. The inclusion of BBC did not affect VFA production in this study, which is in line with Zimmerly and Weiss [4], who found that biotin supplementation at 20 mg/d did not affect the molar percentages of rumen VFA. Meanwhile, Shah et al. [26] reported that betaine supplementation (15 g/d) in dairy cow feed increased the total VFA and acetic acid proportion but decreased the propionic acid proportion; therefore, it is possible that betaine is metabolized in the rumen and converted to acetate [16]. Wang et al. [17] noted that betaine in the rumen could be metabolized into acetate and increase the concentration of acetate at high-dose supplementation levels (100 g/d).

In this experiment, BUN and glucose concentrations were affected by the C:R ratio, which was lower in cattle fed with the 60C:40R ratio diet than in cattle fed with the 70C:30R ratio diet. Lower BUN concentrations in cattle fed with the 60C:40R ratio diets were due to reduced protein intake, which reflects protein degradation in the rumen. Wanapat et al. [2] reported that increasing the concentrate proportion in the diet from 20% to 80% resulted in a linear increase in the BUN concentration (5.8 mg/dL to 7.8 mg/dL).

Hall et al. [15] demonstrated that supplementation with betaine did not elevate glucose levels in dairy cows, except in those subjected to heat stress while receiving high-dose betaine supplementation (114 mg/kg BW). Spears et al. [8] found that blood glucose concentrations were higher in the chromium-supplemented groups than in the control (no chromium) groups of dairy cows. In contrast, insulin concentrations were higher in the control groups than in the chromium-supplemented groups. Therefore, it is impossible to say whether chromium plays a role in animal glucose production. Zimmerly and Weiss [4] demonstrated that concentrations of blood glucose and insulin were not affected by biotin supplementation. Biotin is a key coenzyme involved in gluconeogenesis in the liver [5]. Because insulin governs glucose homeostasis, increased blood glucose concentration encourages insulin synthesis, which then pushes glucose into cells. As a result, high glucose concentrations are rare.

Intuitively, an increased insulin concentration resulted in a decreased glucose concentration (r = 0.42, *p* < 0.05), as also observed by Zimmerly and Weiss [4]. High-producing dairy cows of which the diets were supplemented with biotin experienced an increase in gluconeogenesis, presumably in the liver. The glucose concentration obtained in this study was in line with that of Chen et al. [36], who reported that there was no significant difference in the plasma concentrations of glucose after biotin supplementation at 20 mg/d and 40 mg/d. Ferreira and Weiss [5] observed that liver pyruvate carboxylase mRNA was more abundant and its enzymatic activity was increased by biotin supplementation. In contrast, a report by Stahlhut et al. [37] showed that chromium supplementation could reduce plasma glucose concentrations in growing and finishing steers. Insulin may regulate glucose transport by stimulating the translocation of GLUT 4 from an intracellular membrane pool to the plasma membrane in adipocytes and muscle cells [38]. Kneeskern et al. [13] reported that chromium supplementation (3 mg/d) in feedlot steers could increase insulin concentrations, which supports the observation that supplementing chromium to heifers increased insulin sensitivity [9,10]. Supporting these outcomes, previous studies have reported that supplemental chromium enhanced insulin sensitivity parameters in lactating cattle receiving excessive concentrate and net energy intake [39]. Chromium is a critical component of the glucose tolerance factor, which facilitates the action of insulin on cells, and chromium supplementation has been shown to enhance glucose metabolism in ruminants [10]. According to the findings described here, the inclusion of BBC in the diet of beef cattle could promote the more efficient use of glucose. It was assumed that when supplemented with biotin and chromium, the liver process of gluconeogenesis or glucose transportation, along with increasing insulin activity and insulin sensitivity, would help to improve glucose utilization by enhancing energy and cellular function. In humans, Campbell [40] has shown that using a combination of chromium and biotin enhances glucose uptake. Recently, Turgut et al. [41] found that supplementing diets with chromium and biotin increased serum glucose and lipid levels, as well as PPAR-, IRS-1, and NF-B protein expression levels in the liver and muscle of exercise-trained rats, with the greatest efficacy resulting from their combined administration.

### 4.3. Nitrogen Balance

Nitrogen absorption and nitrogen retention were affected by the C:R ratio. The 70C:30R diet showed higher nitrogen absorption and retention than the 60C:40R diet. Similarly, Suksathit et al. [31] also found that the nitrogen balance was positive and did not differ according to dietary treatments, whereas nitrogen absorption and retention increased as the roughage ratio of hay decreased. Bunting et al. [28] reported that supplemental chromium picolinate did not have any significant influence on the nitrogen balance. However, the nitrogen intake and balance in buffalos is higher at 1.5 mg Cr/kg DM [27]. Our results share a number of common features with those of Kumar et al. [29] who found that dietary chromium supplementation had no effect on nitrogen balance in buffalo calves.

### 4.4. Energy Partitioning and Energy Efficiency

The energy partitioning results of this study demonstrated that neither energy intake nor output were affected by the C:R ratio or BBC supplementation. The digestible energy in the treatment diet was similar to that provided by the control diet. The methane energy loss (6.5% GE intake) was calculated according to the IPCC [22] and was used to derive metabolizable energy values. The metabolizable energy of all of the diets was also unaffected by the C:R ratio or BBC level. The methane energy loss in Thai feedlot native cattle in this trial was supported by reports from Kongphitee et al. [32] and Subepang et al. [42], who found that methane energy emission was 5.91% of GEI intake and 6.25% of GE intake in Thai native cattle, respectively. However, according to another study, Thai native cattle emit 9.04% of their GE intake of methane energy [43]. This variation could be attributable to the type of feed used and the amount of GE intake. In addition, energy efficiency in terms of DE/GE, ME/GE, and ME/DE was consistent with the previous results in Thai native cattle that were fed with diets containing 50% to 80% concentrate [32,42,43].

### 4.5. Weight Gain, Feed Conversion Ratio, Feed Efficiency, and Nutrient Utilization Efficiency

In this study, the weight gained by Thai feedlot native cattle fed with the control diet was 452 g/d, which was close to the initial expectation of 500 g/d. Beef cattle that were fed with diets supplemented with BBC at a rate of 6 g/kg gained more weight (305 g/d) on average than did the beef cattle fed the control diet. Moreover, the weight gain among the cattle supplemented with BBC at 6 g/kg tended (*p* = 0.10) to exceed that gained by the cattle supplemented with BBC at 3 g/kg. Kongphitee et al. [32] reported that their Thai native cattle gained from 391 to 569 g/d when fed with fermented total mixed rations that provided 1.1–1.9-fold more ME for maintenance. However, the weight gain of Thai native cattle varies greatly according to feeding level [44].

From the results obtained in this study, the BBC supplementation groups exhibited decreased FCR and increased feed efficiency. According to the literature, there is no conclusive evidence as to the growth rate in beef cattle that are fed with diets supplemented with one of the single ingredients in BBC. Wang et al. [20] reported that the supplementation of betaine (0.6 g/kg DM) did not affect DMI but increased average daily gain and decreased FCR in bulls. In addition, Kneeskern et al. [15] found that chromium supplementation (3 mg/d) did not affect ADG or FCR in feedlot steers. Biotin (20 mg/d) was given to crossbred cattle by De Silva et al. [45]; however, there was no significant change in their growth rates.

The efficiency of energy and protein utilization was significantly lower in the control than other treatments. In relation to the nutrient requirements for the maintenance and weight gain of Thai native cattle based on the equation reported by WTSR [19], the cattle receiving the treatment diets had energy and protein intakes that were 12% and 11% below the requirement, respectively, whereas the cattle fed with the control diet had intakes that were 16.8% and 26.0% above the requirement, respectively. Dong et al. [46] found that the energy required for maintenance was not constant, but increased as feed intake increased. Cattle fed with treatment diets probably required less energy for maintenance than cattle fed with the control diet, resulting in more energy being available for growth and greater weight gain. Suebpang et al. [42] reported that increasing the feed amount improves ME utilization by reducing the proportion of ME required for maintenance, thereby increasing the proportion dedicated to growth.

## 5. Conclusions

This study showed that BBC supplementation and the C:R ratio had no interactive effect on digestibility, fermentation, nitrogen balance, energy balance, or growth performance of Thai feedlot native beef cattle. BBC supplementation increased weight gain and energy and protein utilization efficiency compared with control treatment. Further studies on the effect of BBC at the tissue level should be conducted.

## Figures and Tables

**Table 1:** The ingredients in the concentrate and the chemical composition of the concentrate, rice straw, and BBC used in the experiment.

Item	Concentrate	Rice Straw	BBC
Ingredients, % DM			
Cassava chip	35.0		
Corn meal	10.0		
Rice bran	10.0		
Palm kernel cake	19.0		
Cassava pulp, dried	14.0		
Soybean meal	8.0		
Urea	1.8		
Sulfur	0.2		
Salt	0.5		
Dicalcium phosphate	1.0		
Premix	0.5		
Chemical composition			
Dry matter (DM), %	95.4	96.4	97.9
	------------------- % DM ------------------		
Organic matter (OM)	94.8	88.9	56.5
Crude protein (CP)	16.1	3.7	7.5
Ether extracts (EE)	3.0	0.9	1.5
Neutral detergent fiber (NDF)	29.7	80.8	-
Acid detergent fiber (ADF)	13.8	49.3	-
Ash	5.2	11.0	43.6
Energy content, MJ/kg DM			
Gross energy (GE)	16.9	16.0	-
Digestible energy (DE)	15.7	8.3	-
Metabolizable energy (ME)	12.8	6.8	-

Premix (per 1 kg) composed of vitamin A: 10,000,000 IU; vitamin E: 70,000 IU; vitamin D: 1,600,000 IU; Fe: 50 g; Zn: 40 g; Mn: 40 g; Co: 0.1 g; Cu: 10 g; Se: 0.1 g; I: 0.5 g.

**Table 2:** Feed intake and apparent digestibility in beef cattle fed with different concentrate-to-roughage ratio diets and different levels of BBC supplementation.

Item	Control	60C:40R		70C:30R		SEM	Contrast (*p*-Value)		
		3BBC	6BBC	3BBC	6BBC		C vs. T	A	B
Dry matter intake, kg/d									
Concentrate	2.59 ^a^	2.14 ^b^	2.17 ^b^	2.53 ^a^	2.48 ^a^	0.04	**	**	0.73
Rice straw	1.08 ^a^	1.37 ^b^	1.36 ^b^	1.01 ^a^	1.02 ^a^	0.03	*	**	0.95
BBC	0.0 ^a^	0.01 ^b^	0.022 ^c^	0.012 ^b^	0.022 ^c^	0.001	**	0.54	**
Total	3.67	3.53	3.55	3.57	3.52	0.06	0.08	0.94	0.87
Concentrate proportion	0.70 ^a^	0.61 ^b^	0.61 ^b^	0.71 ^a^	0.70 ^a^	0.003	**	**	0.54
%BW	3.07 ^a^	3.00 ^b^	2.97 ^b^	2.99 ^b^	3.00 ^b^	0.02	*	0.55	0.49
g/kg W^0.75^	101.5 ^a^	98.4 ^a,b^	97.4 ^b^	98.1 ^b^	98.3 ^b^	0.75	**	0.68	0.62
Nutrient intake, kg/d									
OM	3.4	3.3	3.3	3.3	3.3	0.06	*	0.67	0.80
CP	0.5 ^a^	0.4 ^b^	0.4 ^b^	0.5 ^a^	0.4 ^a^	0.01	**	**	0.68
EE	0.09 ^a^	0.08 ^b^	0.08 ^b^	0.09 ^a^	0.09 ^a^	0.002	*	**	0.56
NDF	1.6 ^a,b^	1.8 ^b^	1.7 ^b^	1.6 ^a^	1.6 ^a^	0.03	0.72	**	0.79
ADF	0.89 ^a^	0.97 ^b^	0.97 ^b^	0.85 ^a^	0.84 ^a^	0.02	0.34	**	0.88
Apparent digestibility, %									
DM	68.4	65.9	68.8	69.7	67.6	1.19	0.78	0.30	0.78
OM	71.8	69.6	72.1	72.8	71.2	1.06	0.76	0.31	0.67
CP	63.9 ^a^	59.4 ^b^	64.4 ^a^	66.1 ^a^	65.6 ^a^	1.27	0.97	**	0.10
EE	86.5	85.7	84.4	88.5	85.9	1.38	0.83	0.14	0.18
NDF	46.5	47.3	51.4	48.9	45.2	1.96	0.44	0.27	0.93
ADF	32.2	35.9	40.7	36.0	30.6	2.42	0.21	0.06	0.90

Control = 70C:30R ratio without BBC supplementation, C vs. T = compared the control treatment to other treatments, A = the effect of concentrate-to-roughage ratios, B = the effect of BBC supplementation. BW = body weight, SEM = standard error of the mean. ^a,b,c^ Means in the same row with different superscripts were significantly different (*p* < 0.05).* (*p* < 0.05), ** (*p* < 0.01).

**Table 3:** Rumen parameters and blood metabolites in beef cattle fed with different concentrate-to-roughage ratio diets and different levels of BBC supplementation.

Item	Control	60C:40R		70C:30R		SEM	Contrast (*p*-Value)		
		3BBC	6BBC	3BBC	6BBC		C vs. T	A	B
Rumen fermentation end-products									
Ruminal pH	6.74	6.78	6.78	6.68	6.76	0.05	0.90	0.23	0.45
NH_3_-N, mg/dL	11.8	11.3	12.8	13.1	11.5	0.74	0.68	0.74	0.96
Total VFA, mM	92.7	81.9	80.1	92.3	114.2	10.06	0.96	*	0.34
Acetic acid (A), mol/100 mol	60.9	58.8	61.0	60.8	58.4	1.10	0.34	0.76	0.93
Propionic acid (P), mol/100 mol	22.6	24.1	23.6	23.4	25.5	0.98	0.17	0.58	0.47
Butyric acid, mol/100 mol	16.5	16.9	15.4	15.9	16.1	1.10	0.78	0.85	0.57
A:P ratio	2.69	2.55	2.67	2.73	2.42	0.16	0.57	0.83	0.56
Blood metabolites									
BUN, mg/dL	8.93	7.33	6.73	7.67	8.80	0.55	0.05	*	0.63
Glucose, mg/dL	92.2	86.5	87.7	90.8	91.4	1.56	0.11	*	0.58
Insulin, µIU/mL	1.68	1.95	1.96	1.78	2.09	0.12	0.08	0.89	0.24
Glucose-to-insulin ratio, mg/µIU	0.56 ^a^	0.47 ^b^	0.47 ^b^	0.52 ^a,b^	0.46 ^b^	0.03	**	0.43	0.31

Control = 70C:30R ratio without BBC supplementation, C vs. T = comparing the control treatment to other treatments, A = the effect of concentrate-to-roughage ratios, B = the effect of BBC supplementation, BUN = blood urea nitrogen, ^a,b^ Means in the same row with different superscripts were significantly different (*p* < 0.05). * (*p* < 0.05), ** (*p* < 0.01).

**Table 4:** Nitrogen balance in beef cattle fed different concentrate-to-roughage ratio diets and different levels of BBC supplementation.

Item	Control	60C:40R		70C:30R		SEM	Contrast (*p*-Value)		
		3BBC	6BBC	3BBC	6BBC		C vs. T	A	B
Nitrogen intake, g/d	70.0 ^a^	60.8 ^b^	61.5 ^b^	68.5 ^a^	67.2 ^a^	1.09	**	**	0.77
Nitrogen excretion, g/d									
Feces	25.0 ^a^	24.3 ^a^	21.5 ^b^	22.9 ^a,b^	22.7 ^a,b^	0.78	*	0.89	0.07
Urine	15.2	12.0	12.1	15.4	12.7	1.20	0.13	0.13	0.31
Total	40.2 ^a^	36.4 ^a,b,c^	33.6 ^c^	38.3 ^a,b^	35.4 ^b,c^	1.31	*	0.18	*
Nitrogen absorption, g/d	45.0 ^a^	36.5 ^b^	39.9 ^b^	45.5 ^a^	44.5 ^a^	1.32	*	**	0.38
Nitrogen retention, g/d	29.8 ^a,b^	24.5 ^b^	27.9 ^a,b^	30.2 ^a^	31.8 ^a^	1.65	0.53	*	0.16
Nitrogen absorption, % of nitrogen intake	64.2 ^a^	59.8 ^b^	64.7 ^a^	66.3 ^a^	65.6 ^a^	1.28	0.96	*	0.12
Nitrogen retention, % of nitrogen intake	42.0	39.5	45.0	43.7	46.1	2.30	0.50	0.22	0.09

Control = 70C:30R ratio without BBC supplementation, C vs. T = comparing the control treatment to other treatments, A = the effect of concentrate-to-roughage ratios, B = the effect of BBC supplementation, SEM = standard error of the mean. ^a,b,c^ Means in the same row with different superscripts were significantly different (*p* < 0.05). * (*p* < 0.05), ** (*p* < 0.01).

**Table 5:** Energy partitioning in beef cattle fed different concentrate-to-roughage ratio diets and different levels of BBC supplementation.

Item	Control	60C:40R		70C:30R		SEM	Contrast (*p*-Value)		
		3BBC	6BBC	3BBC	6BBC		C vs. T	A	B
Energy intake, MJ/d	61.1	59.4	59.9	60.4	60.1	0.87	0.24	0.53	0.90
Energy excretion, MJ/d									
Feces	19.1	19.1	17.1	17.1	17.7	0.65	0.09	0.28	0.34
Urine	1.7	2.3	2.3	1.9	1.9	0.40	0.41	0.32	0.98
Total	20.8	21.4	19.5	19.0	19.6	0.81	0.31	0.18	0.45
Digestible energy intake, MJ/d	42.0	40.3	42.8	43.4	42.3	1.05	0.89	0.24	0.49
Methane energy ^1/^, MJ/d	3.97	3.86	3.90	3.93	3.90	0.06	0.25	0.50	0.90
Metabolizable energy, MJ/d	36.3	34.1	36.6	37.5	36.6	1.02	0.91	0.13	0.48
Energy efficiency									
DE/GE	0.69	0.68	0.70	0.71	0.70	0.01	0.44	0.24	0.52
ME/GE	0.60	0.57	0.60	0.61	0.60	0.01	0.91	0.13	0.45
ME/DE	0.86	0.85	0.85	0.86	0.87	0.01	0.42	0.15	0.62

Control = 70C:30R ratio without BBC supplementation, C vs. T = comparing the control treatment to other treatments, A = the effect of concentrate-to-roughage ratios, B = the effect of BBC supplementation, GE = gross energy, DE = digestible energy, ME = metabolizable energy, CP = crude protein, SEM = standard error of the mean. ^1/^ Methane energy (MJ/d) = 0.065 × GEI (IPCC, 2007).

**Table 6:** Body weight change and feed conversion ratio and feed efficiency of beef cattle fed with different concentrate-to-roughage ratio diets and different levels of BBC supplementation.

Item	Control	60C:40R		70C:30R		SEM	Contrast (*p*-Value)		
		3BBC	6BBC	3BBC	6BBC		C vs. T	A	B
Initial BW, kg	114.8	110.6	112.1	112.5	109.6	2.00	0.14	0.89	0.72
Final BW, kg	124.3	124.4	127.3	126.4	126.1	1.45	0.29	0.81	0.37
Weight changed, kg	9.5 ^a^	13.8 ^ab^	15.3 ^b^	13.9 ^a,b^	16.5 ^b^	1.42	*	0.66	0.17
Weight gain, g/d	452.5 ^a^	658.1 ^a,b^	727.6 ^b^	660.0 ^a,b^	787.6 ^b^	67.69	*	0.55	0.10
Feed conversion ratio, kg feed/kg gain									
	8.9 ^a^	5.5 ^b^	5.2 ^b^	5.5 ^b^	4.6 ^b^	0.70	**	0.68	0.46
Feed efficiency, kg gain/kg feed									
	0.12 ^a^	0.19 ^a,b^	0.21 ^b^	0.19 ^a,b^	0.23 ^b^	0.02	*	0.80	0.28
Nutrient utilization efficiency									
Energy, MJ/d									
Requirement ^1/^	31.7 ^a^	37.8 ^a,b^	40.2 ^b^	38.1 ^a,b^	41.9 ^b^	2.08	**	0.64	0.16
Intake	36.3	33.3	35.5	36.6	35.3	1.02	0.32	0.16	0.68
Efficiency ^2/^	0.87 ^a^	1.18 ^b^	1.21 ^b^	1.10 ^b^	1.23 ^b^	0.06	**	0.76	0.47
Protein, g/d									
Requirement ^1/^	354.0 ^a^	430.4 ^a,b^	457.4 ^b^	431.2 ^a,b^	479.2 ^b^	25.06	**	0.66	0.16
Intake	437.5 ^a^	380.3 ^b^	384.4 ^b^	428.0 ^a^	419.8 ^a^	6.85	**	**	0.76
Efficiency	0.81 ^a^	1.17 ^b^	1.24 ^b^	1.04 ^b^	1.16 ^b^	0.06	**	0.29	0.32

Control = 70C:30R ratio without BBC supplementation, C vs. T = comparing the control treatment to other treatments, A = the effect of concentrate to roughage ratios, B = the effect of BBC supplementation, ME = metabolizable energy, CP = crude protein, SEM = standard error of the mean. ^1/^ ME requirement (kJ/kg W^0.75^) = 486.3 × ADG + 486.3 kJ/kg W^0.75^ and CP requirement (g/kg W^0.75^) = 0.38 × ADG + 5.03 g/kg W^0.75^, where ADG (g/kg W^0.75^) (WTSR, 2010) ^2/^ Efficiency = energy or protein intake/energy or protein requirement. ^a,b^ Means in the same row with different superscripts were significantly different (*p* < 0.05). * (*p* < 0.05), ** (*p* < 0.01).
